# Early P2X7R-dependent activation of microglia during the asymptomatic phase of autoimmune encephalomyelitis

**DOI:** 10.1007/s10787-018-0528-3

**Published:** 2018-09-12

**Authors:** Tomasz Grygorowicz, Lidia Strużyńska

**Affiliations:** Laboratory of Pathoneurochemistry, Department of Neurochemistry, Mossakowski Medical Research Centre, Polish Academy of Sciences, 5 Pawińskiego str., 02-106 Warsaw, Poland

**Keywords:** Neuroinflammation, Purinergic receptors, Multiple sclerosis, EAE, Iba-1, Brilliant Blue G

## Abstract

Microglia-mediated neuroinflammation accompanies many central nervous system (CNS) diseases, including multiple sclerosis (MS), and is strongly dependent on the purinergic P2X7 receptor. The nature of the inflammatory response in MS is studied for decades indicating, that proinflammatory microgliosis is involved in advanced stages of MS and is associated with active tissue damage and neurological dysfunctions. Evidence on the role of microgliosis in initial stages of the disease is scarce. Thus, in the present study, we investigated the time course of microglial activation in rat brain subjected to experimental autoimmune encephalomyelitis (EAE) which is the animal model of MS. We show that activation of microglia occurs in brains of immunized rats at a very early stage of EAE, well before the development of neurological symptoms of the disease. Enhanced immunoreactivity of microglia/macrophage-specific protein Iba-1, together with morphological features of microgliosis, was identified beginning at day 4 post immunization. Concomitantly, microglial expression of P2X7R was also examined. Moreover, our results reveal that administration of Brilliant Blue G, an antagonist of P2X7R, delays the onset of the disease and partially inhibits development of neurological symptoms in EAE rats. Blockage of P2X7R significantly reduces activation of microglia as confirmed by decreased Iba-1 immunoreactivity and suppresses neuroinflammation in EAE rat brains, as indicated by decreased protein levels of investigated proinflammatory cytokines: IL-1β, IL-6 and TNF-α. Our results indicate that microglia are involved in inducing neuroinflammation at a very early stage of MS/EAE via a P2X7R-dependent mechanism.

## Introduction

Multiple sclerosis (MS) is a progressive demyelinating inflammatory disease of the central nervous system (CNS). In MS, as well as in its well-characterized animal model, experimental autoimmune encephalomyelitis (EAE), peripheral autoreactive T-cells specific for myelin antigens infiltrate the CNS and initiate an inflammatory reaction resulting in dysfunction of the blood–brain barrier (BBB), demyelination and neurodegenerative changes (Lassmann 2018). Local inflammation is fueled by T cell-derived cytokines which further activate both microglia and astroglia. Excessive activation of glial cells has deleterious consequences in the form of the release of signaling molecules involved in inflammation and neurodegeneration such as cytokines, reactive oxygen/nitrogen species and glutamate (Dheen et al. 2007).

One of the functions of microglia is to receive signals sent by injured cells and to react by removing cellular remnants in the process of phagocytosis (Monif et al. 2010). It has been reported that CNS-resident microglia are inert (Amadio et al. 2017) or less active (Barnett and Prineas 2004) during the initial stage of MS/EAE and participate rather in later phases of the disease, contributing to the release of proinflammatory cytokines and the removal of myelin debris within plaques. In MS patients, positive correlation was found between activation of microglia and destruction of myelin sheaths (Lassmann et al. 2007). Increased reactivity of microglia was also identified in EAE mice in the symptomatic phase correlating with inflammatory infiltration of parenchyma (Ayers et al. 2004). Without fail, activation of microglia accompanies development of both MS and EAE. Moreover, regulation of microglia activity may influence the outcome of the disease. Inhibition of macrophages/microglia at the onset of EAE symptoms (i.e., day 7 post immunization) was found to significantly decrease the progression of neurological deficits (Bhasin et al. 2007). It does not mean, however, that activated microglia only exert an adverse proinflammatory impact. Phagocytosis of myelin debris in MS lesions, expression of anti-inflammatory and tissue repair factors by activated microglia are essential processes to promote remyelination (Luo et al. 2017; Napoli and Neumann 2010). As it has been reported, functions of activated microglia are complex and the final effect depends on the timing and the form (proinflammatory M1/anti-inflammatory M2) of activation (Gao and Tsirka 2011).

Extracellular ATP is a strong signaling molecule in the CNS responsible for intercellular communication (Cotrina et al. 2000; Inoue et al. 2007) and acts as a natural agonist of an array of ionotropic (P2X) and metabotropic (P2Y) purinergic receptors. Among the P2X type of ATP-gated ion channels, the P2X7 receptor is widely expressed in brain cells (Sperlagh et al. 2006) and plays a substantial role in numerous brain pathologies, including MS (Sperlagh and Illes 2014).

Purinergic signaling, particularly P2X7R-mediated signaling, plays a pivotal role in activation and proliferation of microglia as shown in cultures of hippocampus tissue (Monif et al. 2009). Numerous reports have also showed that over-expression/over-activation of this receptor underlies a microglia-induced inflammatory reaction which is associated with the release of inflammatory and bioactive substances (Suzuki et al. 2004; Inoue 2002). Released inflammatory mediators drive a self-propagating cycle via an autocrine mechanism which further promotes neuroinflammation and neurodegeneration (Monif et al. 2010). P2X7R-induced depolarization and associated K^+^ efflux (Riedel et al. 2007) leads to activation of a protein complex known as the inflammasome, via a caspase 1-dependent mechanism. The activated inflammasome causes proteolytic cleavage of the inactive form of IL-1β (pre-IL-1β, 30-35 kDa) and secretion of a mature form of interleukin IL-1β (18 kDa) (Mariathasan et al. 2006) which initiates the inflammatory cascade (Mingam et al. 2008). Evidence also exists that P2X7R is involved in the release of IL-6 (Solini et al. 1999). Thus, this receptor significantly contributes to the inflammatory process.

In spinal cords of MS patients, P2X7 was found to be upregulated in plaques formed around blood vessels mainly in activated microglial cells/macrophages (Amadio et al. 2017). Moreover, P2X7R-deficient mice were found to be more resistant to EAE than wild-type mice exhibiting reduced neuroinflammation and axonal damage (Sharp et al. 2008).

With the knowledge that activated microglia-dependent inflammation is implicated in the pathogenesis of MS, we focused on the response of this pool of glial cells during the course of EAE. We have addressed the question of whether microglia are activated in the pre-onset phase of EAE, and release proinflammatory cytokines and whether this activation is P2X7R dependent. We started the analysis at the preclinical stage (day 2–4 p.i.), well before the first neurological deficits appeared, and concentrated on obtaining evidence of activation of microglia and protein expression of cytokines such as interleukin 1β (IL-1β), interleukin 6 (IL-6), and tumor necrosis factor (TNF-α). The potential role of the P2X7R purinergic receptor in inducing activation of microglia in brains of immunized rats was verified using Brilliant Blue G (BBG), a selective antagonist of P2X7R.

## Materials and methods

### Animal model of EAE

Female Lewis rats weighing 190–200 g and sourced from the Animal House at the Mossakowski Medical Research Centre of the Polish Academy of Sciences (Warsaw, Poland) were used throughout the experiments. Spinal cords for rat immunization were isolated from guinea pigs obtained from Charles River Laboratories International Inc., Germany. Experimental procedures involving animals were performed in accordance with EU Directive 2010/63/EU and approved by the local Experimental Animal Care and Use Committee (Approval no. 48/2011). The number of Ethics Committee approval (48/2011) consists of the decision number (48) and the year of decision (2011).

EAE was induced according to our standard protocol. Briefly, rats were immunized with inoculum containing spinal cord of guinea pig homogenized in PBS and emulsified in Freund’s complete adjuvant with 2 mg/mL of *Mycobacterium tuberculosis* (H37Ra) (Difco, Detroit, MI, USA). A single intradermal injection of 100 μL of inoculum was administered into each footpad of animals. A selective antagonist of P2X7R, Brilliant blue G (BBG), was dissolved in saline solution and administered daily to EAE rats in a dose of 50 mg/kg b.w. starting from day 0 until day 6 post immunization via a catheter implanted into the internal jugular vein. Appropriate control groups were also used. The vehicle control group received saline instead of BBG. The dose of the antagonist was selected based on the literature (Carmo et al. 2014; Geraghty et al. 2017) and our own preliminary studies.

The condition of the animals was monitored daily. Disease progression was assessed based on the developing neurological deficits scored as described previously (Grygorowicz et al. 2016) using the following scale: 0- no symptoms; 1- limp tail; 2- hind limb weakness; 3- hind limb paralysis; 4- ascending paralysis; and 5- moribund (Kerschensteiner et al. 2004). Animals were sacrificed at different time-points of the disease: in the asymptomatic (4 d.p.i.) and symptomatic phases (12 d.p.i.). To analyze the temporal profile of cytokine proteins, EAE rats were sacrificed at different time-points (2, 4, 6, 8 days) during the asymptomatic phase and in the symptomatic phase (12 d.p.i.). Neither EAE nor EAE + BBG animals were housed longer, until recovery phase of the disease.

After decapitation and rapid preparation, the brains were washed in 50 mM phosphate buffer, pH 7.4, frozen in liquid nitrogen and stored at − 80° C.

### Western blot analysis

To prepare homogenates, the forebrains were homogenized in 50 mM phosphate buffer, pH 7.4 containing 10 mM EGTA, 10 mM EDTA, 0.1 mM PMSF and 100 mM NaCl in the presence of a protease inhibitor cocktail (1 μg/mL leupeptin, 0.1 μg/mL pepstatin and 1 μg/mL aprotinin). The protein concentration in homogenates was measured according to the method of Lowry et al. (1951) using bovine albumin as a standard.

Samples of 20–40 μg of protein/lane were subjected to SDS-PAGE in 10% acrylamide mini-gels, transferred further onto nitrocellulose membranes (Hybond-ECL, Amersham, UK) and examined for the expression of proinflammatory cytokines. Blots were incubated with primary antibody: anti-IL-1β (1:500, R&D System, MN, USA), anti-IL-6 (1:250; R&D System, MN, USA), anti-TNF-α (1:500, R&D System, MN, USA). Monoclonal anti-actin antibody (specific towards α, β, γ forms of the actin) was used as internal standard (1:1000; MP Biomedicals, Warsaw, Poland). Thereafter, the respective secondary anti-goat or anti-mouse HRP-conjugated antibody (Sigma Aldrich, Inc., St. Louis, MO, USA) was applied at a dilution of 1:10,000. Bands were visualized using the ECL kit and quantified by densitometric analysis using ImageScanner III (GE Healthcare) and the ImageQuant TL v2005 program.

### Immunohistochemical procedure and microscopic analysis

Animals (four per group) were anesthetized with a lethal dose of Narcotan–Halothanum (Zentiva, Prague, Czech Republic) and perfused through the heart with phosphate-buffered saline (PBS) at pH 7.4 and subsequently with 250 mL of ice-cold fixative (4% paraformaldehyde; Sigma-Aldrich, Inc., St. Louis, MO, USA; in PBS). After post-fixation in the same fixative for 1.5 h, brains were cryoprotected overnight in 10% sucrose in PBS, followed by 20% sucrose for 2 days and 30% sucrose for 4–5 days. Thereafter, frozen tissue was cut into 40-µm sections. The sections were collected free-floating in PBS, pH 7.4 with 0.1% sodium azide and then stored at − 20 °C in antifreeze medium (30% sucrose, 60% glycol ethylene, 0.05 M PBS buffer, pH 7.4).

Immunostaining was performed using primary anti-Iba-1 (1:500; Abcam, Cambridge, GB) and anti-P2X7R antibodies (1:200; Alomone Labs, Jerusalem, Israel), and further with secondary antibody conjugated with Alexa Fluor (1:200; Invitrogen Corp., Carlsbad, CA, USA). To control immunostaining specificity, the primary antibody was omitted from the incubation mixture. Brain sections were mounted on silane slides, air-dried and coverslipped under Vectashield Mounting Medium (Vector). Images were obtained using a confocal laser scanning microscope (Zeiss LSM 510) and processed using the Zeiss LSM 510 software package v. 3.2. Mean fluorescence intensity on micrographs was measured on the whole image area using ZEN Black Edition 2012 software.

### Statistical analysis

The results are expressed as the mean ± SD from *n* experiments. Evaluation of significant differences among groups was performed using one-way analysis of variance (ANOVA) followed by the post hoc Dunnett’s test. *p* < 0.05 was considered significant.

## Results

### The course of EAE

Animals were monitored daily for signs of the disease. The first neurological deficits in immunized rats appeared at day 11, peaked at day 13 and then recovered. According to the five-point scale of presentation of neurological symptoms (Kerschensteiner et al. 2004), the symptoms were demonstrated as progressive paralysis of tail and hind limbs and reduction of physical activity, as well as a significant loss of body weight.

Administration of P2X7R antagonist (BBG) delayed the onset of the disease by 2 days, and reduced maximal disease score from 3 to 1.5 points (Table 1).

**Table 1:** Clinical parameters of animals subjected to EAE and animals simultaneously treated with P2X7R antagonist (BBG)

Parameter	EAE	EAE + BBG
Maximal cumulative index (score)	3 ± 0.5	1.5 ± 0.3*
Duration of inductive phase (days)	11 ± 1.0	13 ± 0.5

The values represent the mean ± SD from eight animals in each group

*p < 0.05 values significantly different from EAE

### Early activation of brain microglia in the pre-onset of EAE is P2X7R dependent

**Fig. 1:**
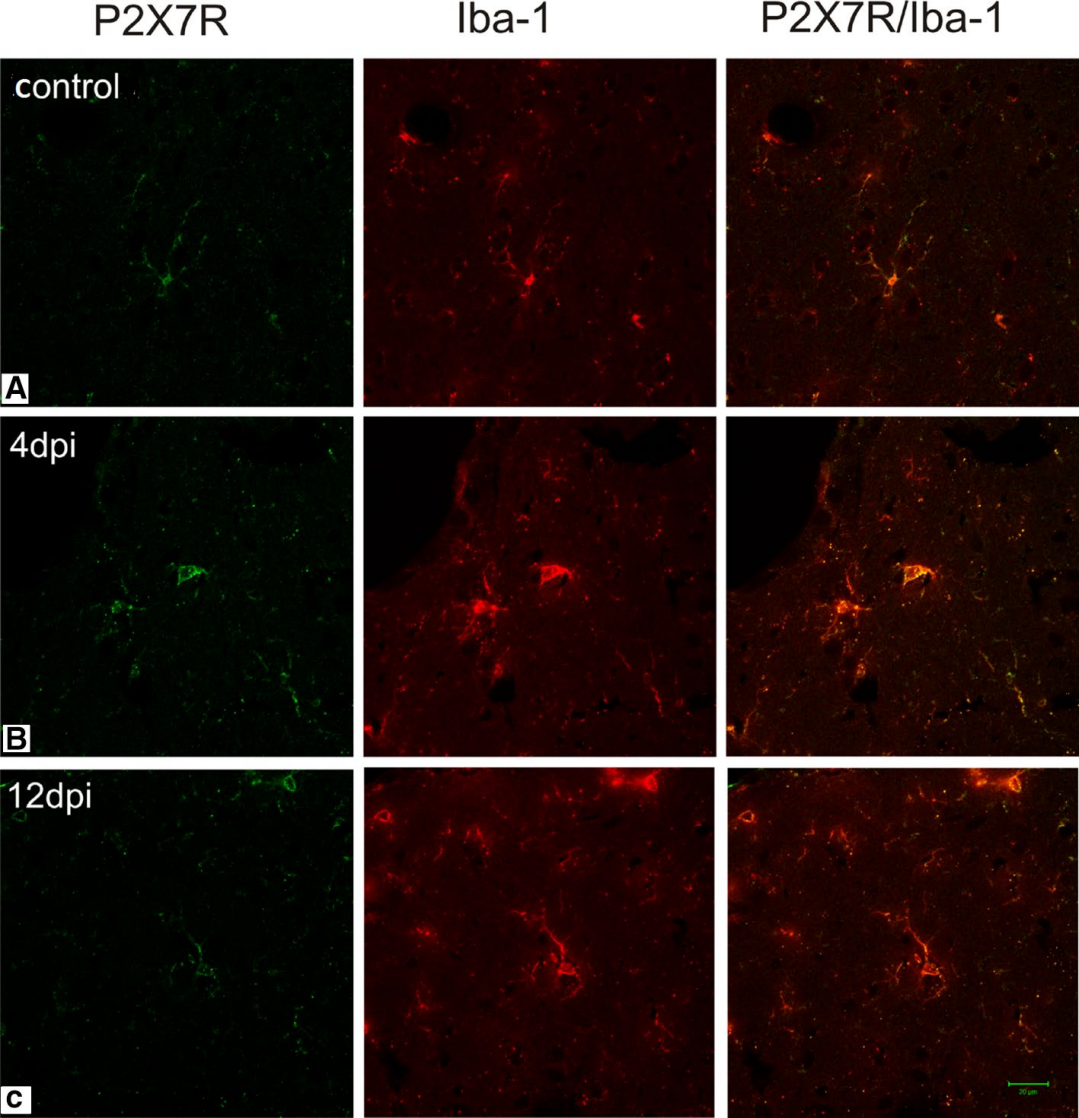
Expression of P2X7R in microglial cells. Immunofluorescence image of double immunostaining Iba-1/P2X7R in forebrain of control (**a**) and EAE rats in asymptomatic (4 d.p.i.) (**b**) and symptomatic (12 d.p.i.) (**c**) phases of the disease. Activated Iba-1/P2X7R-positive cells are seen in immunized rats (**b**, **c**). Images are representative for each of four animals

**Fig. 2:**
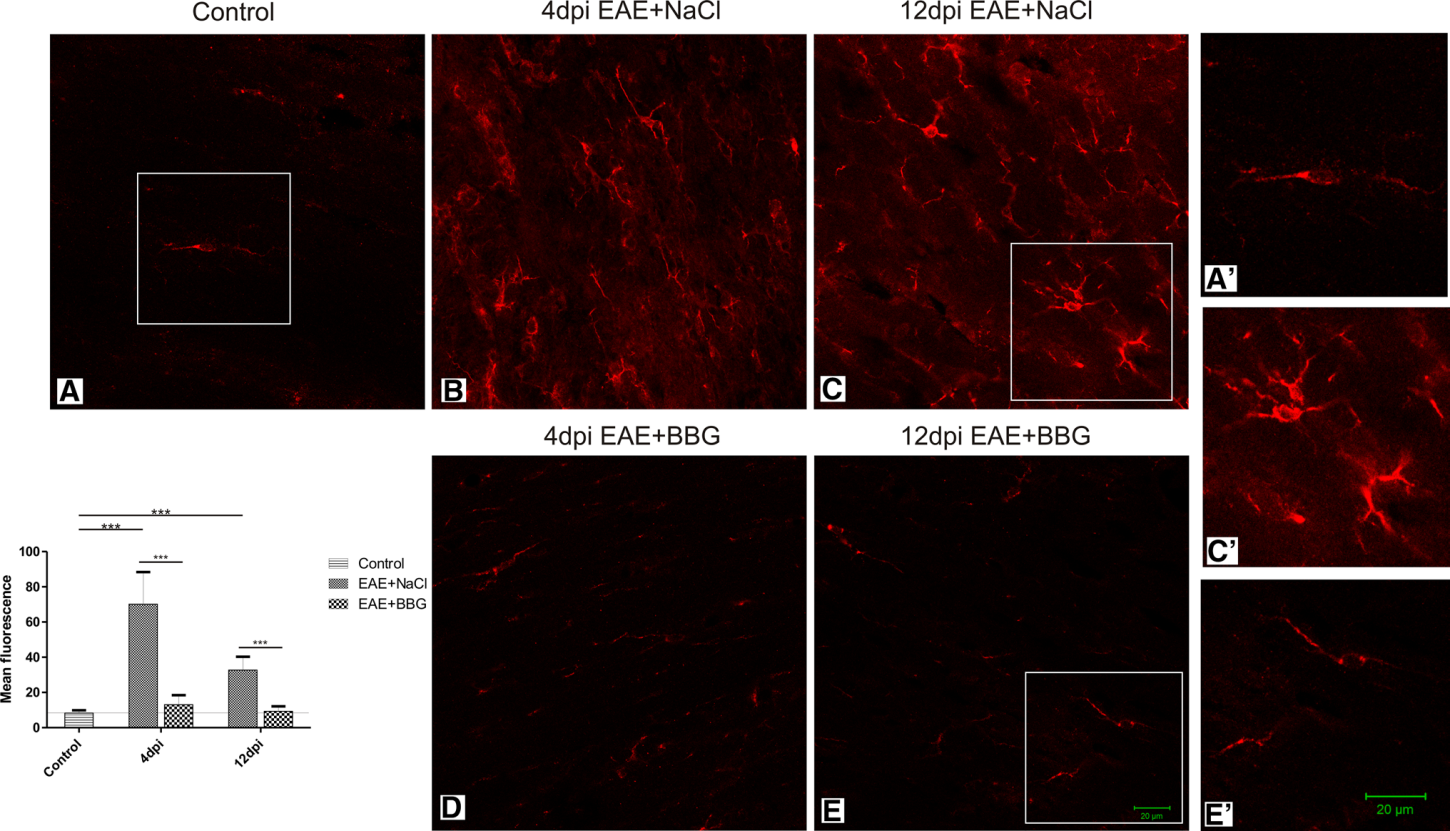
Activation of microglia during the course of EAE is suppressed by the P2X7R antagonist. Immunofluorescence image of Iba-1immunoreactive cells in forebrain of control (**a**), EAE rats (**b**, **c**) and EAE rats treated with BBG (**d**, **e**) in asymptomatic (4 d.p.i.) and symptomatic (12 d.p.i.) phases. Insets present magnification of resting (**a**′, **e**′) and activated (**c**′) microglial cells. The graph indicates the mean intensity of the fluorescence signal. ***p < 0.001 compared with control non-immunized rats or EAE rats (one-way ANOVA with the post hoc Dunnett’s test). Images are representative for each of four animals

Microgliosis is the known hallmark of symptomatic MS/EAE. The results of our study revealed the occurrence of early activation of this pool of brain cells. Images obtained by confocal microscopy showed enhanced immunoreactivity of the microglial/macrophage-specific protein Iba-1 in brains of EAE rats in both the asymptomatic and symptomatic phases of the disease, i.e., at day 4 and 12 p.i., respectively (Figs. 1b, c; 2b, c). Over-expression of this protein is associated with microglial activation (Imai and Kohsaka 2002). Additionally, microglial cells were found to exhibit feature characteristic of the activated state such as shortening, thickening and increased numbers of cytoplasmic processes, as well as large amounts of cytoplasm (Figs. 1b, c; 2b, c, c′).

Double immunostaining with anti-P2X7R showed the presence of this receptor on microglial cells in both non-activated (control) and activated states (EAE—4 and 12 d.p.i.) (Fig. 1). After administration of BBG to immunized rats, the immunoreactivity of Iba-1 markedly declined in the early phase of EAE and remained reduced also in the symptomatic phase (12 d.p.i.) as demonstrated by a measurement of mean fluorescence intensity (Fig. 2d, e and graph), indicating that blockade of P2X7R attenuates activation of microglia.

### Blockage of P2X7R decreases the level of proinflammatory cytokines in EAE rat brains

During the course of EAE, the protein levels of cytokines rise significantly. IL-1β, IL-6 and TNF-α are considered proinflammatory cytokines and are useful markers of ongoing inflammatory processes. The relative protein concentration (calculated against the internal standard) of all examined cytokines was found to be significantly higher in EAE rats compared to the control non-immunized animals.

**Fig. 3:**
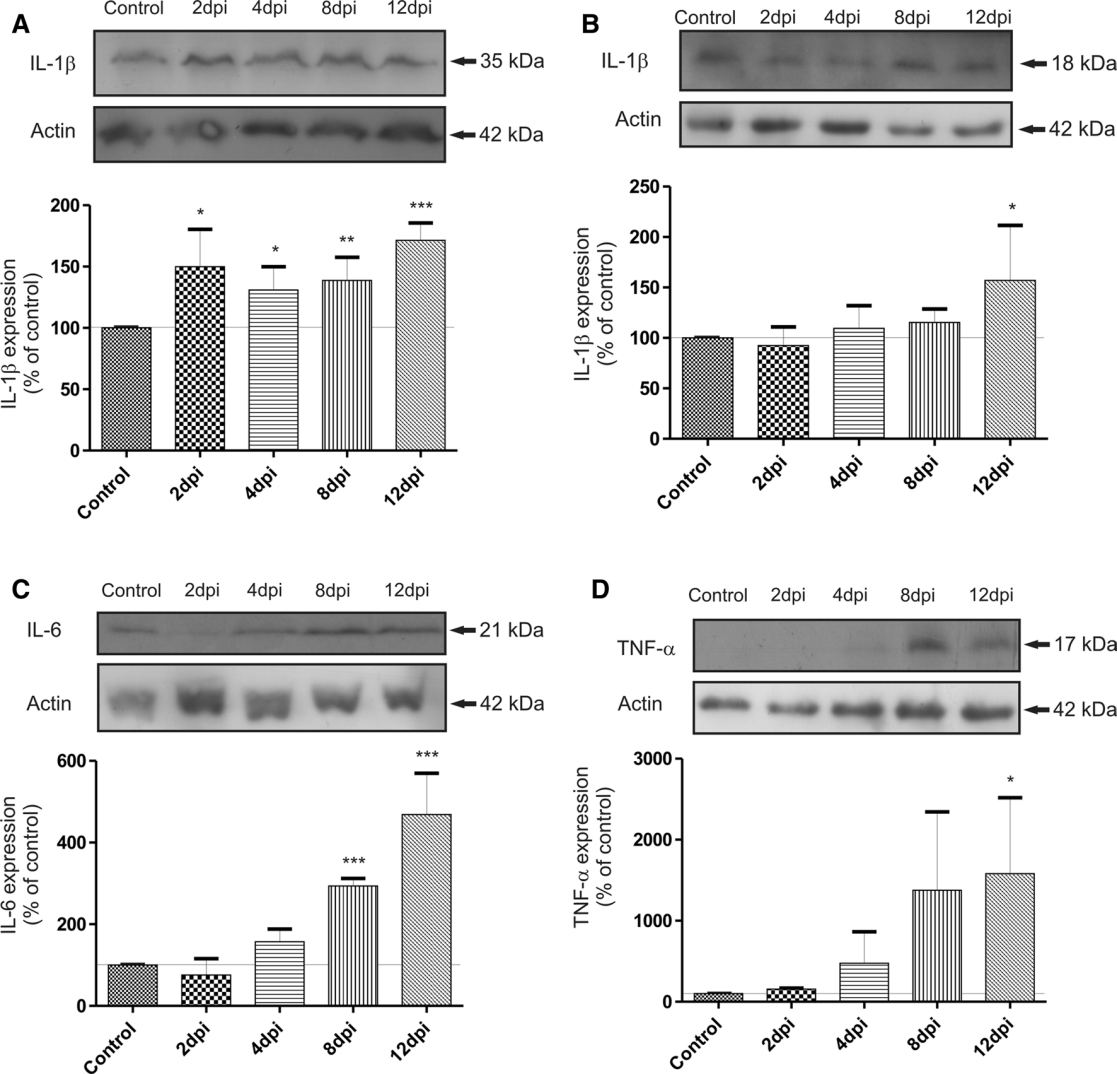
Representative immunoblots showing the protein expression of proinflammatory cytokines **a** pro-IL-1β, **b** IL-1β, **c** IL-6, **d** TNF-α in brain homogenates obtained from control and EAE rats at different times post immunization (2-12 d.p.i.). The graph indicates the results of densitometric measurements of four independent immunoblots in each group of animals with samples including four distinct forebrains and expressed as a percentage of control. The relative density was measured against actin as an internal standard. *p < 0.05; **p < 0.01 and ***p < 0.001 compared with control (one-way ANOVA with the post hoc Dunnett’s test)

The most important interleukin, whose expression is controlled by a P2X7R-dependent mechanism, is IL-1β. This cytokine is present in the cell in inactive form (35 kDa) and is cleaved by proteolytic enzyme to generate the active cytokine (18 kDa). Protein expression of the precursor form was found to increase significantly (by about 40–50%) in the early phase (2–8 d.p.i.) of EAE (*p < 0.05 or **p < 0.01 vs. non-immunized control) and to remain elevated during the symptomatic phase of the disease (12 d.p.i.) (***p < 0.001) (Fig. 3a). Protein expression of an active form of IL-1β was found to increase gradually, reaching a maximal level significantly elevated from the control value by about 60%, in the symptomatic phase (12 d.p.i.) (Fig. 3b). Concomitantly with activation of microglia, we also noted a significant increase in the relative protein concentration of IL-6 by about 368% (**p < 0.001 vs. non-immunized control) and TNF-α by about 1380% (*p < 0.05 vs. non-immunized control) in the symptomatic phase of EAE (12 d.p.i.) (Fig. 3c, d).

**Fig. 4:**
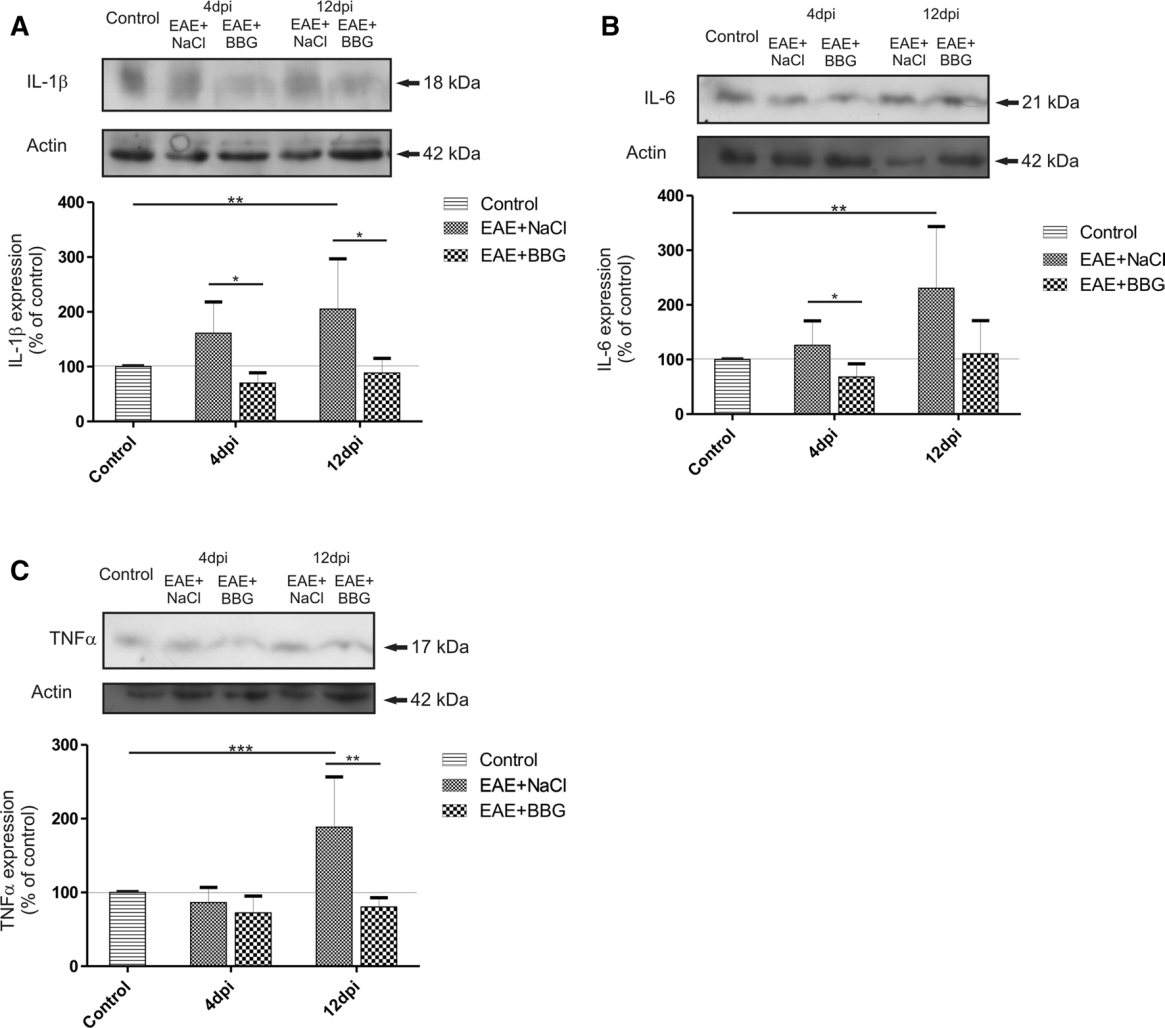
Representative immunoblot showing the expression of **a** IL-1β; **b** IL-6; **c** TNF-α protein in asymptomatic (4 d.p.i.) and symptomatic (12 d.p.i.) phases of the disease in brain homogenates of control and immunized rats treated or not with BBG. The graph indicates the results of densitometric measurements of 3–4 independent immunoblots performed using 3–4 distinct forebrains and expressed as a percentage of control. The relative density was calculated against actin as an internal standard. *p < 0.05, **p < 0.01 and ***p < 0.001 vs. EAE group or control non-immunized group (one-way ANOVA with the post hoc Dunnett’s test)

BBG-administered EAE rats showed significant lowering of all examined cytokines which begins in the asymptomatic (IL-1β and IL-6) and lasts in the symptomatic phase of the disease (IL-1β and TNF-α) (Fig. 4a–c). The relative levels of cytokines were not only diminished compared to EAE rats but even returned to the control values.

## Discussion

### Early activation of microglia during the course of EAE

In the present study, we investigated the temporal profile of activation of microglia and the changes in levels of proinflammatory cytokines in brains of rats subjected to EAE. We show that microglia undergo activation early after immunization, in the pre-onset phase of the disease, well before the development of neurological symptoms, as confirmed by the increased immunoreactivity of Iba-1 (microglia/macrophage marker) and altered morphology of microglial cell characteristic of the activated state. Under physiological conditions, microglia are present in a quiescent state with relatively low volumes of cellular cytoplasm and multiple thin processes. Under pathological stimuli, these cells become enlarged with retracted processes (Monif et al. 2010). It is known that microglia become activated during MS/EAE and may be involved in the development and expansion of the disease. This phenomenon has been related to the removal of myelin debris in active demyelinating plaques in the late phase of the disease (Amadio et al. 2017), but activated microglial/macrophage cells were also observed in inflammatory non-demyelinating areas where they persisted for the whole course of the disease (Mikita et al. 2011; Gao and Tsirka 2011).

Upon activation, microglia secrete various inflammatory substances, including cytokines and chemokines. As discussed by Gao and Tsirka (2011), the intensity, timing profile and type of microglia activation (M1 vs. M2) may have an significant impact on the outcome of the disease.

Under the conditions of EAE applied in the current study, the relative protein levels of all examined cytokines (IL-1β, IL-6 and TNF-α) were found to peak very early in temporal coincidence with enhanced immunoreactivity of Iba-1, thus confirming that microglia is engaged in inducing inflammation. However, microglial are antigen-presenting cells involved in cross-reactions with other cells, including astrocytes (Luo et al. 2017). Thus, astrocyte-induced release of proinflammatory cytokines is not excluded, since we have found previously that likewise astrocytes are activated early during the course of EAE (Grygorowicz et al. 2016).

### P2X7R antagonist prevents proinflammatory activation of microglia

The presence of both quiescent and activated P2X7R on microglial cells in the nervous system has been reported (Parvathenani et al. 2003; Yiangou et al. 2006; Kobayashi et al. 2011). Regarding MS/EAE, the role of this receptor has been described in context of death of oligodendroglial cells at the point of prevalence of neurological symptoms (Matute et al. 2007) and has been related to activation of astroglia (Grygorowicz et al. 2016; Amadio et al. 2017). However, increased expression of P2X7R in demyelinating plaques of post mortem MS spinal cords was shown to occur within activated microglial cells/macrophages (Amadio et al. 2017). The results of our study confirmed localization of P2X7R in microglia (Fig. 2).

P2X7R is essential for induction of microglial activation and proliferation, which plays a pivotal role in the neuroinflammatory cascade (Monif et al. 2010). The relationship between the expression of P2X7R and microglia-dependent release of proinflammatory cytokines has been confirmed (Ferrari et al. 1997; Inoue 2002). Activation of P2X7R leads to formation of the inflammasome and maturation and subsequent release of IL-1β. Therefore, enhanced levels of IL-1β protein may reflect the stimulation of this receptor. In MS cases, this interleukin has been shown to be released from microglia/macrophages via P2X7R-dependent induction of cyclooxygenase-2 and downstream pathogenic mediators (Yiangou et al. 2006).

Our results show the contribution of P2X7R in early proinflammatory activation of microglia during EAE. Under the conditions of the study, blockage of the P2X7 receptor by its selective antagonist (BBG) influences the state of microglial cells. Immunofluorescence experiments reveal inhibition of microgliosis in brains of rats subjected to EAE with concomitant administration of BBG. We found that microglial cells lose morphological signs of activation and the intensity of Iba-1 immunoreactivity returns to the control value. Mean fluorescence measured in microscopic images was found to be sevenfold (asymptomatic phase) or threefold (symptomatic phase) lower compared to the EAE animals which did not receive BBG (Fig. 3, graph). Moreover, blockage of P2X7R diminishes the microglia-dependent release of proinflammatory cytokines such as IL-1β, IL-6 and TNF-α. The BBG-dependent decrease of the cytokines was found to be constant during the course of the disease. The cytokine protein levels were close to control values in both asymptomatic and symptomatic phases of EAE. The results associated with administration of BBG indicate that P2X7R is certainly involved in activation of microglia at a very early stage of EAE.

It is noteworthy that in the CNS, P2X7R is also expressed on astrocytes (Oliveira et al. 2011) and, as we have previously shown, activated astrocytes overexpress this receptor starting from the very early asymptomatic phase of EAE (Grygorowicz et al. 2016). Obviously, administration of BBG significantly reduces activation of this pool of glial cells. Astrocytes, coupled functionally with neurons on one side and pericytes, the basement membrane (BM) and capillary endothelial cells (ECs) on the other side, represent an active part of the neurovascular unit (NVU) which provides the functional basis of cerebral microvessels (Figley and Stroman 2011). Thus, perivascular astrocytes are “the first line” of glial cells contacting the inflammatory immune cells as they infiltrate nervous tissue from the blood. Therefore, considering the results of the current study in the context of our previous data and existing knowledge, we cannot exclude the possibility that activation of microglia is secondary to astroglial activation and occurs via cytokine/chemokine crosstalk with astroglia.

It was also found in the present and previous study (Grygorowicz et al. 2016) that administration of BBG delays the onset of the disease and attenuates neurological deficits, as well as significantly improves the general appearance of immunized animals. Both astroglia and microglia express P2X7R and may contribute to neuroinflammatory processes. Therefore, we assume that both pools of cells should be considered equally important in the pathogenesis of MS/EAE, since they share the common pathological mechanisms, which may be the target of pharmacological intervention.

### Conclusion

Our study demonstrates early changes in microglia in the pre-onset stage of EAE, thus providing actual data on the time-dependent contribution of these cells in pathological processes during the disease. We show here that the early activation of microglial cells in brains of rats subjected to EAE starts well before the development of neurological symptoms. Simultaneously, administration of P2XR antagonist (BBG) significantly suppresses microglial activation, delays onset of the disease in animals and partially alleviates neurological deficits. Our data suggest involvement of the P2X7-mediated purinergic signaling pathway in the mechanism of early microgliosis during the course of EAE, which by driving and sustaining neuroinflammation, can be a significant player in development of the disease.
